# Treatment of Chancres

**Published:** 1845-01

**Authors:** 


*Treatment of Chancres.*—Mr. T. Bartlett, an army surgeon, treats chancre in the following manner, his object being to obtain cicatrization as speedily as possible, in order to prevent absorption of the venereal virus, and consequent secondary symptoms:—He applies a dry stick of lunar caustic lightly over the whole surface of the chancre, and then dry lint; on the falling off of the slough, which sometimes takes place in twenty-four, but more generally in forty-eight hours, dry calomel is generally sprinkled over every part of the sore, previously well-washed; after which a strip of lint, sufficiently long to surround the penis, is applied, after it has been wetted with cold water. The further treatment consists in applying calomel and wet lint once a day in this way, after the sore has been thoroughly cleansed from all the discharge by lukewarm water. When there is a short prepuce, strips of sticking plaster are required to approximate its extremities to prevent the lint falling off. Whenever the chancre affects any portion of the frœnum, division by the knife is necessary, to prevent the cure being retarded. No constitutional treatment is necessary. Mr. Bartlett states that he has had only one case of secondary symptoms since he adopted this plan of treatment, and in that case it appears to have been owing to neglect on the part of the patient. In another case, that of a man of weakly habit and sickly appearance, sloughing of the cellular membrane of the prepuce supervened, and was successfully treated with poultices, warm fomentations, &c.—*Ibid.*

